# Cerebral Infarction Followed by Myocardial Infarction in a Young Adult with Protein C and S Deficiency

**DOI:** 10.7759/cureus.6665

**Published:** 2020-01-15

**Authors:** Faryal Tahir, Zainab Majid, Taha Bin Arif, Jawad Ahmed

**Affiliations:** 1 Internal Medicine, Dow University of Health Sciences, Karachi, PAK

**Keywords:** myocardial infarction, cerebral infarction, arterial thrombosis, protein c deficiency, protein s deficiency, thrombophilia, dilated cardiomyopathy, stroke, hypercoagulation

## Abstract

Protein C (PC) and protein S (PS) are natural anticoagulants that protect the body against thrombosis, and their deficiency, either inherited or acquired, renders the body to a hypercoagulable state. This leads to venous thromboembolism manifesting as thrombosis, pulmonary embolism and superficial thrombophlebitis among other causes. The involvement of arteries is rare and has been explained by only a few studies. Hence, the presentation of PC and PS deficiencies with stroke and myocardial infarction (MI) is rarely observed, especially in young patients.

We report a case of a 33-year old male with a past medical history of stroke and MI for which no underlying cause was found. He presented now with shortness of breath and left-sided chest pain and after a series of workup, eventually diagnosed as a rare case of PC and PS deficiencies.

## Introduction

Protein C (PC) and protein S (PS) are vitamin K-dependent glycoproteins that provide protection against thrombosis, hence acting as natural anticoagulants [1]. PC is cleaved to form activated protein C (APC) which inactivates clotting factors V and VIII [2]. PS not only acts as a cofactor to APC but also binds to factors Xa and Va, exhibiting impairment of prothrombin activation [2]. The deficiency of these proteins, although rare, can be either inherited or acquired, rendering the patient to a hypercoagulable state. The association of PC and PS deficiencies with venous thromboembolism (VTE) has been well established resulting commonly in deep vein thrombosis, pulmonary embolism (PE) and superficial thrombophlebitis among other causes [3]. However, the involvement of arteries in the setting of PC and PS deficiencies has been found to be significantly lower, making arterial thromboembolism (ATE) and subsequent events like myocardial infarction (MI) and non-hemorrhagic stroke rare complications [4]. To date, a few studies have further investigated and explained this rare association, for example, data published in a 2008 article showed a fivefold increased risk of ATE occurring at a young age [5].

We report a case of a 33-year-old male with a past medical history of stroke and MI for which no underlying cause was found. He presented now with shortness of breath (SOB) and left-sided chest pain and after a series of workup, eventually diagnosed as a rare case of PC and PS deficiencies.

## Case presentation

A 33-year-old male with a significant medical history of hypertension and smoking presented to outpatient department with complaints of left-sided chest pain, SOB and episodic cough. The pain was moderate to severe in intensity, radiating to left arm and neck, continuous in nature, aggravated with movements with no relieving factors. It was not associated with fever, headache, vomiting or abdominal symptoms. The SOB was mostly experienced on walking and occasionally when lying flat. His family history was positive for cardiovascular disease as his father had fatal MI at a young age.

**Figure 1:**
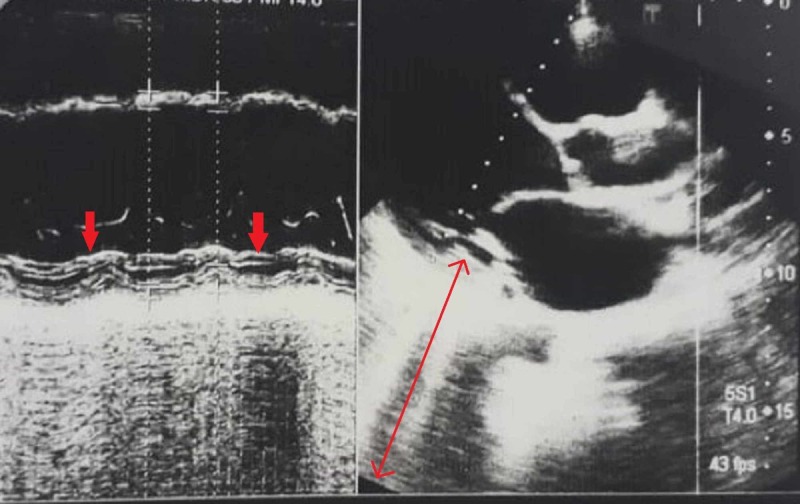
TTE showing concentric LV hypertrophy TTE: transthoracic echocardiogram, LV: left ventricle

Past medical records depicted that he was admitted to a local hospital in 2014 with a diagnosis of right-sided weakness secondary to cerebrovascular accielectrocardiogram dent. The magnetic resonance imaging scans of brain showed an acute infarction of area supplied by the left middle cerebral artery (MCA). Magnetic resonance angiography of the circle of Willis depicted occlusion of the left MCA at the horizontal M1 segment. The extracranial arteries did not show any abnormalities like plaque formation on evaluation by cervical echography. After initial management with intravenous (IV) aspirin, the patient was administered with IV clopidogrel. Long-term anticoagulation was not prescribed since no cause was identified. There were no complications and the patient’s hemiparesis gradually improved during the following months. Later in 2017, he developed SOB with acute retrosternal chest pain. His electrocardiogram (ECG) showed ST-segment elevation in leads V2-V4. Troponin T was raised up to 7,120 ng/L [normal (N)= 0-14]. Transthoracic echocardiogram (TTE) showed moderate left ventricular (LV) systolic dysfunction, concentric LV hypertrophy, regional wall motion abnormities and mild mitral regurgitation (Figure 1). The patient was initially administered with xylocaine, heparin, nitroglycerine, atropine, metoprolol, clopidogrel and loop diuretics. Cardiac catheterization revealed total occlusion of the left anterior descending (LAD) artery for which the patient underwent primary angioplasty to LAD with a drug-eluting stent. After adequate management, the patient was discharged on aspirin, clopidogrel, bisoprolol, rosuvastatin, spironolactone, hydroxyzine and loop diuretics.

On examination, his blood pressure (BP) was 165/90 mmHg, respiratory rate was 24 breaths per minute and heart rate was 70 beats per minute with no arrhythmia. General physical examination revealed palmar erythema, splinter hemorrhages and mild pedal edema. The SOB of New York Heart Association class III along with orthopnea was accompanied by productive cough and hemoptysis. Apex beat could not be palpated while lung fields were found to be clear on auscultation. Neurological examination showed no focal neurological deficits with a Glasgow Coma Scale score of 15/15. Other systemic examinations were insignificant.

Laboratory evaluation on admission revealed hemoglobin of 14.5 g/dL (N=13.8-17.2), total leukocyte count of 7 x 10^9^/L (N=4-11), platelet count of 249 x 10^9^/L (N=150-400), prothrombin time of 10.7 seconds (N=11-13.5), activated partial thromboplastin time of 16.7 seconds (N=26-36) and international normalized ratio of 1.10 (N≤ 1.1). Serum electrolytes were normal, and creatinine was documented to be 0.8 mg/dL (N=0.6-1.2). Total bilirubin, albumin and globulin levels were within the normal range. Lipid profile was also normal with serum cholesterol of 114 mg/dL (N< 200), low-density lipoprotein of 55 mg/dL (N=60-130), high-density lipoprotein of 60 mg/dL (N=60), and triglyceride level of 75 mg/dL (N<150). However troponin I levels were raised up to 0.1 ng/mL (N≤ 0.04).

**Figure 2:**
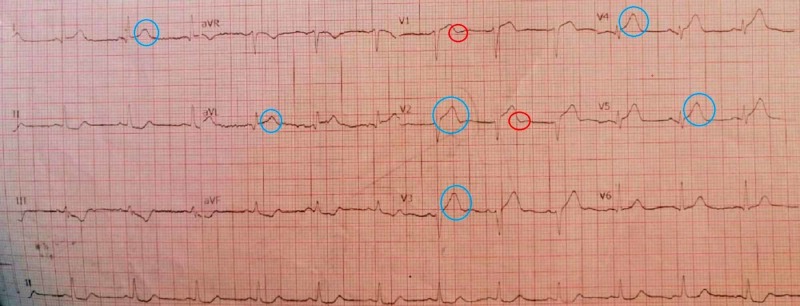
ECG showing ST-segment elevation in leads I, V2-V5 and aVL (blue circle) and T-wave inversion in leads V1-V2 (red circle) ECG: electrocardiogram

**Figure 3:**
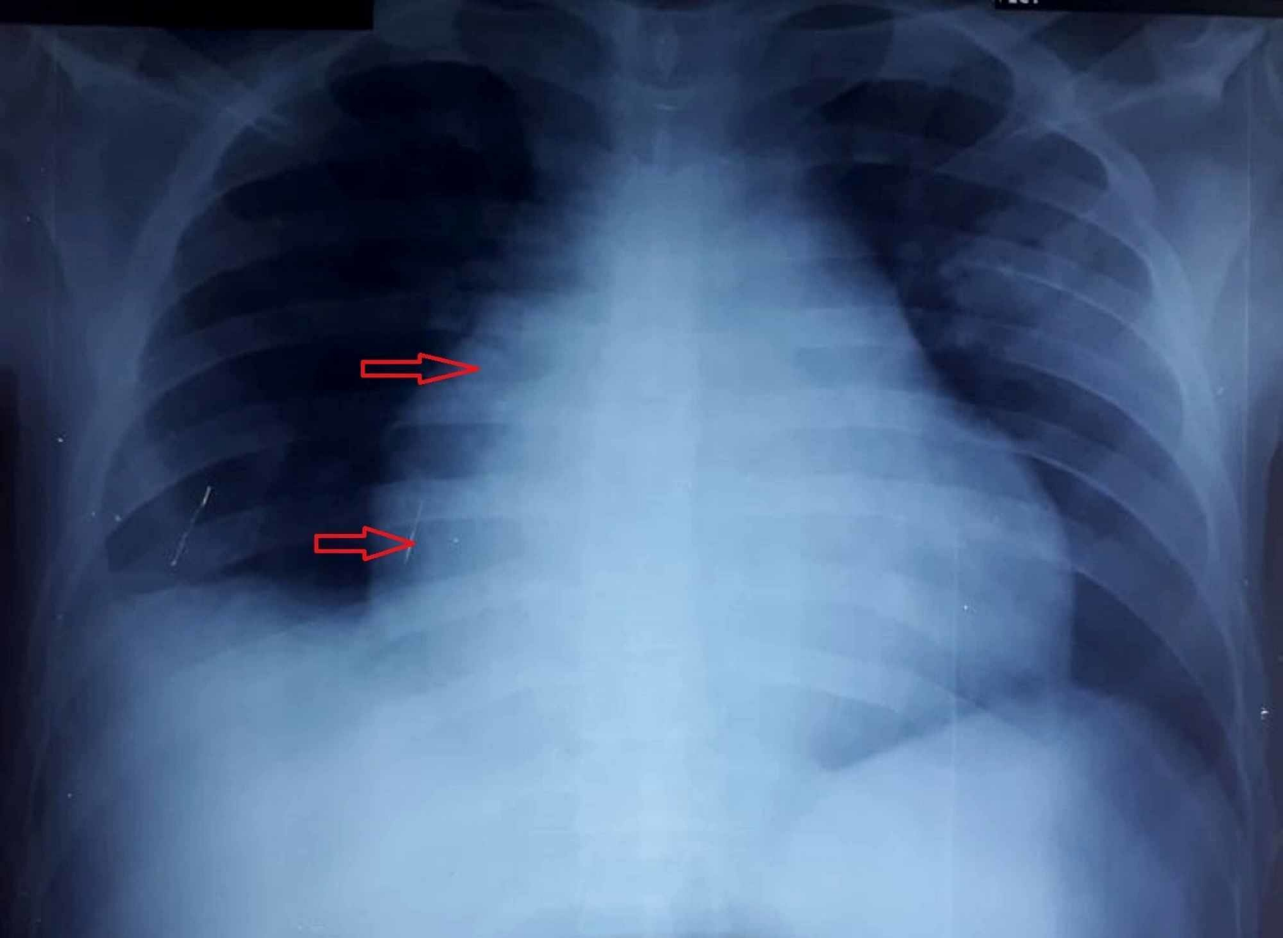
CXR showing enlargement of the cardiac silhouette (red arrows) CXR: chest X-ray

The initial ECG had 1-2 mm ST-segment elevation in leads I, V2-V5 and aVL with ST-segment depression in reciprocal leads (Figure 2). Chest X-ray showed an enlargement of the cardiac silhouette with diffuse pulmonary edema (Figure 3). The patient was initially managed with ascard, atorvastatin, clopidogrel, valsartan, bisoprolol, spironolactone,and omeprazole.

TTE showed dilated LV with moderate dysfunction, akinetic LV apex, apical mid interventricular septum and inferior wall, and an ejection fraction (EF) of 35%. Carotid Doppler and duplex study of neck vessels (common carotid, external carotid, internal carotid and vertebral arteries) were found to be normal. Cardiac catheterization revealed a patent stent and thrombotic occlusion of the LAD. Considering the past history of stroke and MI in this young patient, blood samples were sent for a detailed coagulation profile. The serum homocysteine level was found to be 10 µmol/L (N=4-15), while factor V Leiden was also negative. Deficiency of PC and PS was finally diagnosed; values seen were 41.9% (N=70-123) and 53% (N=70-140), respectively.

Although the patient recovered from SOB and pedal edema, the EF showed no improvement despite adequate antiplatelet therapy. The BP also dropped to 100/70 mmHg. The patient was discharged with anticoagulants, diuretics, beta-blockers and nitrates. The patient was advised for regular follow-ups, but unfortunately, he could not maintain follow-ups.

## Discussion

Stroke is defined as a multiple pathologic process, consequently leading to focal intracranial infarction. Approximately 85% of the strokes are ischemic, whereas only 15% are hemorrhagic, usually occurring in the elderly population. Despite ample investigations, a large number of ischemic strokes affecting the young population are of unknown etiology. Young ischemic stroke requires a cascade of diagnostic evaluations to investigate for an underlying thrombophilic state. Carod et al. studied the subtypes of ischemic stroke and the incidence of thrombophilia in stroke patients from Brazil [6]. They investigated 130 young and 200 elderly patients of thrombophilia with PS deficiency (11.5% vs 5.5%) and PC deficiency (0.76% vs 1%), respectively. It was concluded that patients with stroke of undetermined causes were more frequently preceded by prothrombotic conditions.

Thrombophilic disorders can be classified into two major categories: inherited and acquired. Inherited thrombophilias are rare in young ischemic strokes as occurred in our case. PC, PS and antithrombin III deficiencies and factor V Leiden mutation account for the major causes behind these ischemic conditions. However, among all of them, factor V Leiden mutation is the most common cause, affecting 50% of the cases. But in our patient, factor V Leiden mutation was absent. Contrarily, acquired thrombophilias are relatively more common such as antiphospholipid antibody syndrome. This leads to the rarity of our case where PC and PS deficiencies resulted in the occlusion of the left MCA followed by a focal ischemic stroke.

**Figure 4:**
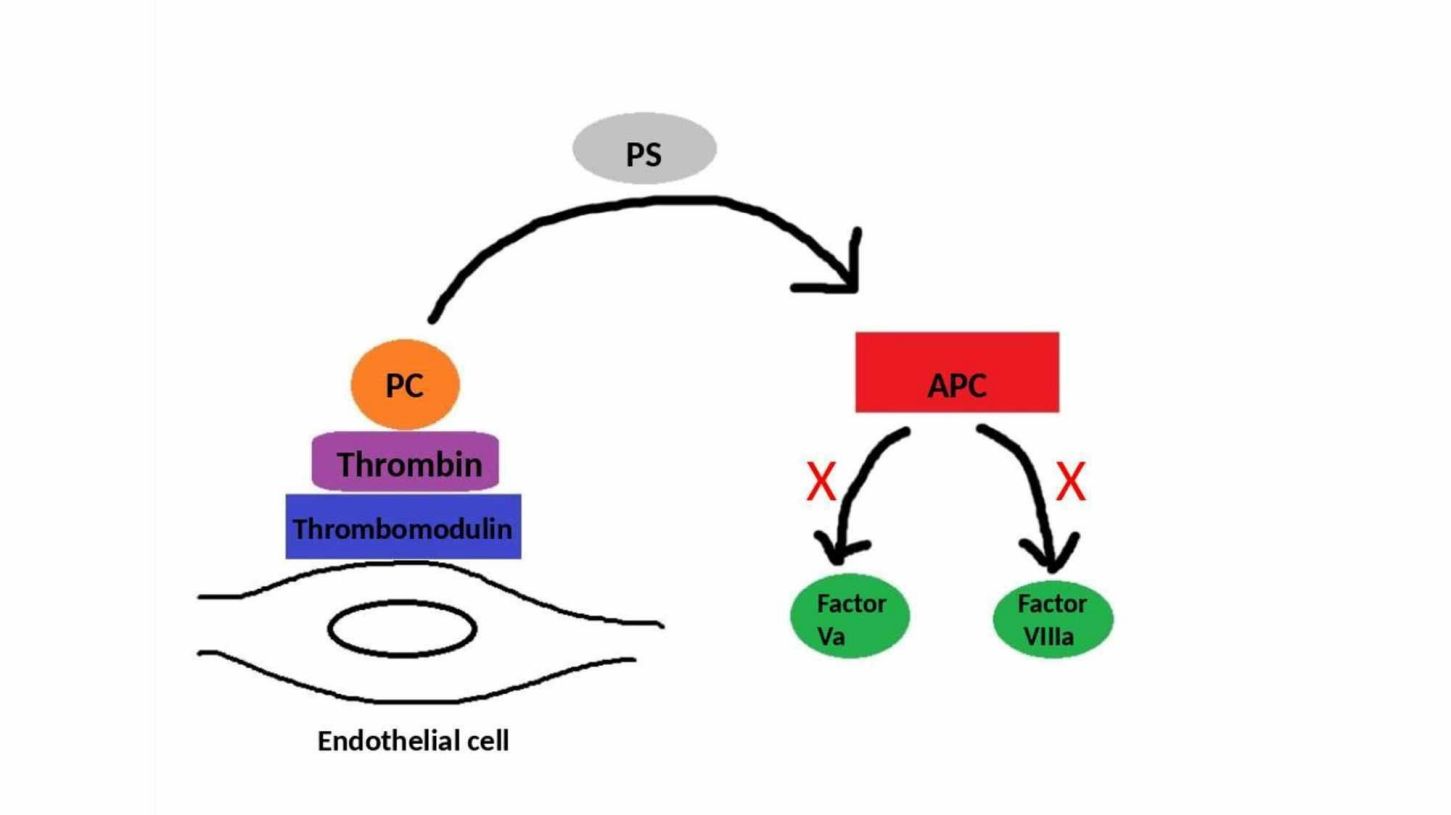
The effect of PC PC (protein C), PS (protein S), APC (activated protein C)

**Figure 5:**
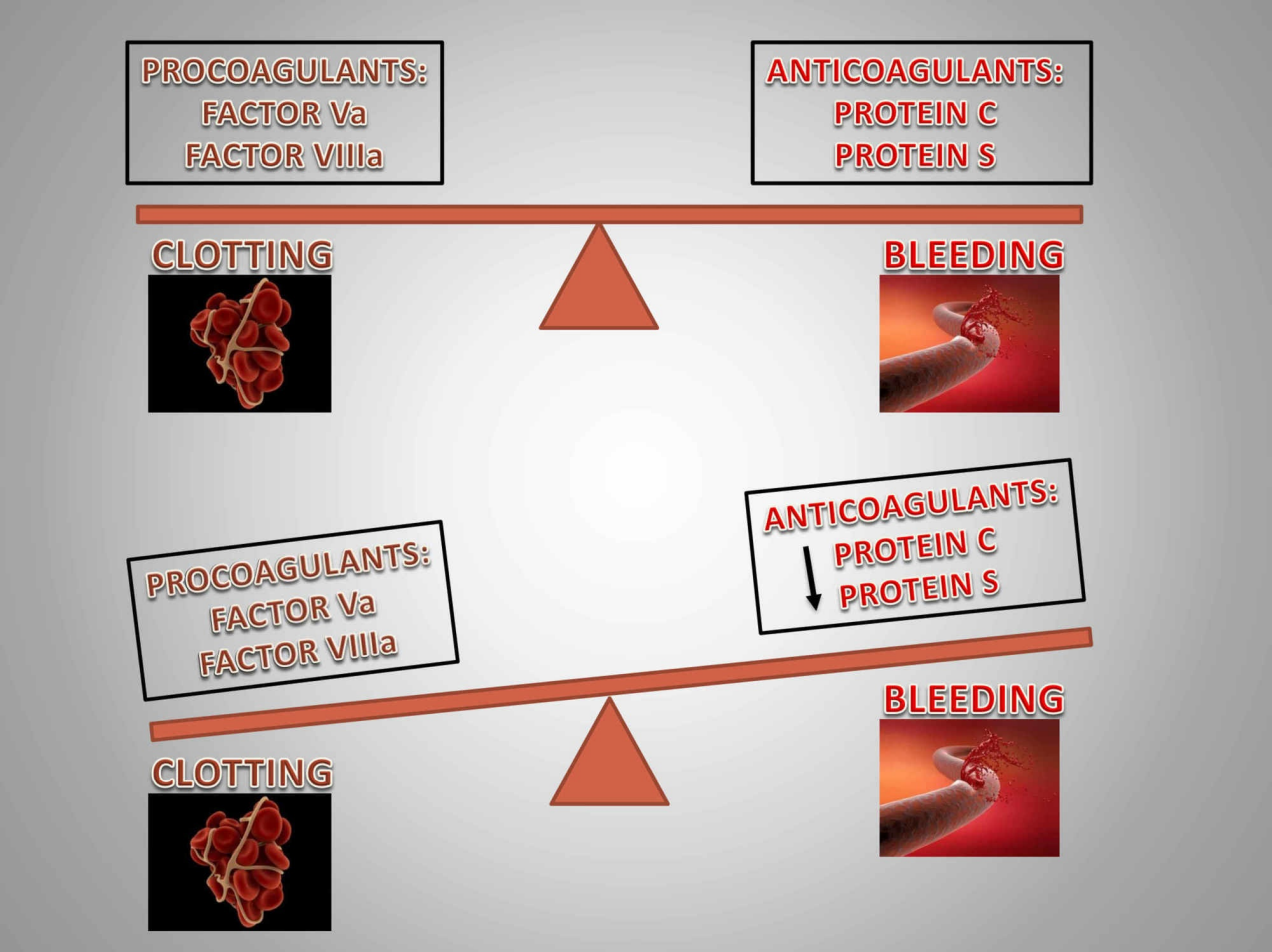
The homeostasis between procoagulants and anticoagulants

PC, a vitamin K-dependent plasma protein, acts as an anticoagulant by proteolytically degrading activated factor V and inactivating factor VIII, thereby facilitating thrombolysis via its fibrinolytic action. PS serves as a cofactor for PC (Figures 4, 5) [7].

An association between PC deficiency and arterial ischemic stroke has been reported [8]. The relevance of VTE or PE secondary to the deficiencies of these natural anticoagulants is well established. However, there are conflicts reported from India as well as other countries limiting the reliability of the association between PC deficiency and arterial ischemic stroke [9,10].

Although our patient recovered from the stroke after adequate management, the exact cause of thrombosis was not ruled out. Three years later, our patient ended up with MI due to thrombotic occlusion of the LAD artery. After being recovered, the patient’s SOB worsened day by day. On further evaluation, a deficiency of PC and PS was diagnosed as the major cause for these events. Our patient will be treated with life-long anticoagulants, as well as aggressive lifestyle modification. 

A study comprising of 337 patients with heterozygous PC deficiency showed arterial thrombosis in only 7.1% of the subjects [11]. A recent work involving 255 patients with ST-elevation MI in <35 years of age, revealed a deficiency of PC in only one of them (0.4%) [12]. A cohort study highlighted arterial thrombotic events in only 8% of the 144 patients with deficiencies of both PC and PS [13]. It further adds up to the rarity of our case as both the events were secondary to arterial occlusion. PC deficiency does not predispose to arterial thrombosis by itself; however, when coupled with smoking or high-risk family history, it is associated with a higher incidence of premature MI as happened in our patient [14]. According to our literature search, there is only one case reported with stroke and MI both occurring secondary to PC and PS deficiencies [15].

## Conclusions

PC and PS deficiencies are probable causes of stroke and MI in a young patient, without any underlying risk factor for coronary or cerebral arterial diseases and should always be considered in unexplained cases of thrombosis. A thorough analysis of these deficiencies is essential for a timely diagnosis and improved outcomes. Evidence of arterial involvement needs further research to establish this life-threatening association. 

## References

[1] Lipe B, Ornstein DL (2011). Deficiencies of natural anticoagulants, protein C, protein S, and antithrombin. Circulation.

[2] Wypasek E, Undas A (2013). Protein C and protein S deficiency-practical diagnostic issues. Adv Clin Exp Med.

[3] Soare AM, Popa C (2010). Deficiencies of proteins C, S and antithrombin and activated protein C resistance-their involvement in the occurrence of arterial thromboses. J Med Life.

[4] Simioni P, Zanardi S, Saracino A, Girolami A (1992). Occurrence of arterial thrombosis in a cohort of patients with hereditary deficiency of clotting inhibitors. J Med.

[5] Mahmoodi BK, Brouwer JLP, Veeger NJGM, van der Meer J (2008). Hereditary deficiency of protein C or protein S confers increased risk of arterial thromboembolic events at a young age: results from a large family cohort study. Circulation.

[6] Carod-Artal FJ, Nunes SV, Portugal D, Silva TV, Vargas AP (2005). Ischemic stroke subtypes and thrombophilia in young and elderly Brazilian stroke patients admitted to a rehabilitation hospital. Stroke.

[7] Clouse LH, Comp PC (1986). The regulation of hemostasis: the protein C system. N Engl J Med.

[8] Kohler J, Kasper J, Witt I, von Reutern GM (1990). Ischemic stroke due to protein C deficiency. Stroke.

[9] Girolami A, Simioni P, Lazzaro AR, Cordiano I (1989). Severe arterial cerebral thrombosis in a patient with protein S deficiency (moderately reduced total and markedly reduced free protein S): a family study. Thromb Haemost.

[10] Sié P, Boneu B, Biermé R, Wiesel ML, Grunebaum L, Cazenave JP (1989). Arterial thrombosis and protein S deficiency. Thromb Haemost.

[11] De Stefano V, Leone G, Micalizzi P, Teofili L, Falappa PG, Pollari G, Bizzi B (1991). Arterial thrombosis as clinical manifestation of congenital protein C deficiency. Ann Hematol.

[12] Rallidis LS, Gialeraki A, Tsirebolos G, Tsalavoutas S, Rallidi M, Iliodromitis E (2017). Prothrombotic genetic risk factors in patients with very early ST-segment elevation myocardial infarction. J Thromb Thrombolysis.

[13] Boekholdt SM, Kramer MH (2007). Arterial thrombosis and the role of thrombophilia. Semin Thromb Hemost.

[14] Bux-Gewehr I, Nacke A, Feurle GE (1999). Recurring myocardial infarction in a 35 year old woman. Heart.

[15] Tiong IY, Alkotob ML, Ghaffari S (2003). Protein C deficiency manifesting as an acute myocardial infarction and ischaemic stroke. Heart.

